# Influence of antioxidant (L- ascorbic acid) on tolbutamide induced hypoglycaemia/antihyperglycaemia in normal and diabetic rats

**DOI:** 10.1186/1472-6823-5-2

**Published:** 2005-03-03

**Authors:** Satyanarayana Sreemantula, Eswar K Kilari, Vishnu A Vardhan, Rajasekhar Jaladi

**Affiliations:** 1 Pharmacology Division, University College of Pharmaceutical sciences, Andhra University, Visakhapatnam, – 530 003, India

## Abstract

**Background:**

Diabetes mellitus is a chronic metabolic disorder characterized by hyperglycaemia. Increased oxidative stress and decreased antioxidant levels are the leading cause of diabetes and diabetic complications. So it is felt that supplementation of antioxidants may be useful in controlling the glucose levels and to postpone the occurrence of diabetic complications. The objective of our study is to find the influence of antioxidant supplementation (L-ascorbic acid) on tolbutamide activity in normal and diabetic rats.

**Methods:**

L- ascorbic acid/tolbutamide/L-ascorbic acid + tolbutamide were administered orally to 3 different groups of albino rats of either sex in normal and diabetic condition. Blood samples were collected from retro-orbital puncture at different time intervals and were analyzed for blood glucose by GOD-POD method. Diabetes was induced by alloxan 100 mg/kg body weight administered by I.P route.

**Results:**

L-ascorbic acid/ tolbutamide produced hypoglycaemic activity in a dose dependant manner in normal and diabetic condition. In the presence of L-ascorbic acid, tolbuatmide produced early onset of action and maintained for longer period compared to tolbutamide matching control.

**Conclusion:**

Supplementation of antioxidants like L-ascorbic acid was found to improve tolbutamide response in normal and diabetic rats.

## Background

Diabetes mellitus is a chronic metabolic disorder characterized by hyperglycaemia. It requires life long treatment with drugs coupled with diet control and exercise. It may be due to decrease in the synthesis of insulin (Type-I diabetes) or due to decrease in the secretion of insulin from β-cells of islets of Langerhans of pancreas (Type-II diabetes). Insulin is the drug of choice in type – I diabetes and sulfonylureas are the drugs of choice in type II. Among sulfonylureas, tolbutamide is the drug of choice for geriatrics because of its short duration of action and lower incidence of hypoglycaemia in early hours of night.

Diabetes is one of the stress related disorder. Diabetic subjects are shown to have increased oxidative stress and decreased antioxidant levels [1-3]. It was also shown that tight control of blood glucose is possible with decrease in oxidative stress [4]. Antioxidants are claimed to work as antistress agents by decreasing oxidative stress. L-ascorbic acid used in therapy for disorders like scurvy produces antioxidant activity. Earlier reports show that the relationship between scurvy and diabetes mellitus indicates the low levels of plasma ascorbic acid in diabetic rats compared to control rats [5,6]. Hence the present study was conducted to find the influence of L-ascorbic acid, a water soluble antioxidant and a free radical scavenger on the hypoglycaemic and antihyperglycaemic activities of tolbutamide in normal and diabetic rats.

## Methods

All animal experiments were performed in accordance with our institutional animal ethics committee. Albino rats of either sex (Mahaveer Enterprises, Hyderabad) weighing between 125 – 175 g were used in the study. They were housed five per cage at temperature 22 ± 2°C and 12/12 h light/dark under controlled environment. Rats were fed with standard pellet diet. (Mahaveer Enterprises, Hyderabad) and water *ad libitum*. They were divided into 3 groups of five each. They were fasted for 18 h prior to the experiment allowing access to water only, and the water was withdrawn during the experiment. Blood samples were collected from the retro-orbital plexus of each rat at 0, 0.5, 1, 1.5, 2, 4, 6 hr after drug administration. Blood glucose levels were determined by using GOD – POD method [7].

Group I received L-ascorbic acid 60 mg / kg body weight, Group II received tolbutamide 20 mg/kg body weight and Group III was given L-ascorbic acid (60 mg / kg body weight) prior to the administration of tolbutamide 20 mg/kg body weight in normal rats. In clinical practice tolbutamide and vitamin C are administered orally hence in our study also this was administered orally.

### Induction of diabetes

Albino rats of either sex weighing between 125 to 175 g were fasted overnight before injection with alloxan. Alloxan monohydrate was dissolved in saline solution and was administered at a dose of 100 mg/kg body weight intraperitonially. Animals were treated with 10% dextrose orally to combat the early phase of hypoglycaemia. Rats showing fasting blood glucose levels above 150 mg/dl were selected for the study. They were divided into 3 groups of five each. Group I received L-ascorbic acid 40 mg/kg body weight and Group II received tolbutamide 20 mg/kg body weight while Group III was given L-ascorbic acid 40 mg/kg prior to tolbutamide administration (20 mg/kg). L-ascorbic acid dose was fixed based on its response, which produced above 40%.

### Statistical analysis

The significance of blood glucose reduction produced by L-ascorbic acid with tolbutamide compared to tolbutamide control was determined by applying students unpaired t-test and the significance is indicated by * mark.

## Results

The presence of L-ascorbic acid upto 20 μg did not interfere with the blood glucose estimation when tested with different quantities in *in vitro* studies. In normal rats L-ascorbic acid at the dose of 60 mg/kg body weight administered orally produced 50.91% blood glucose reduction at 0.5 h and 20 mg/kg body weight of tolbutamide produced 33% at 4 h as peak effects. In the presence of L-ascorbic acid (60 mg/kg), the action of tolbutamide was early in onset and maintained for 6 h. In diabetic rats, oral administration of L-ascorbic acid alone at the dose of 40 mg/kg body weight produced 42.53% blood glucose reduction at 1.5 h and tolbutamide 20 mg/kg body weight produced 45.09 at 4 h. Administration of L-ascorbic acid 40 mg/kg body weight prior to tolbutamide produced antidiabetic activity at 0.5 h and was maintained for 6 h. The percent blood glucose reduction with L-ascorbic acid / tolbutamide/ L-ascorbic acid + tolbutamide in normal rats and diabetic rats were given in table 1 &2.

**Table 1:** Percent blood glucose reduction (Mean ± SEM) with L-ascorbic acid/ tolbutamide / L-ascorbic acid + tolbutamide in normal rats (n= 5)

**Time in hours**	**L-ascorbic acid 60 mg/kg bd.wt.**	**Tolbutamide 20 mg/kg bd.wt.**	**L-ascorbic acid 60 mg/kg bd.wt. + Tolbutamide 20 mg/kg bd.wt.**
0	-	-	-
0.5	50.91 ± 0.49	10.08 ± 1.08	53.57 ± 2.01***
1	20.26 ± 3.14	15.56 ± 1.48	28.70 ± 1.75***
1.5	6.06 ± 1.3	18.75 ± 2.1	23.25 ± 1.72
2	1.65 ± 0.93	22.22 ± 2.13	29.78 ± 2.57
4	-2.83 ± 0.4	33.0 ± 0.69	45.21 ± 2.79**
6	-	10.28 ± 1.02	12.54 ± 2.5

Significance *** P < 0.001, **P < 0.01

**Table 2:** Percent blood glucose reduction (Mean ± SEM) with L-ascorbic acid/ tolbutamide / L-ascorbic acid + tolbutamide in diabetic rats (n = 5)

**Time in hours**	**L-ascorbic acid 40 mg/kg bd.wt.**	**Tolbutamide 20 mg/kg bd.wt.**	**L-ascorbic acid 40 mg/kg bd.wt. + Tolbutamide 20 mg/kg bd.wt.**
0	-	-	-
0.5	18.23 ± 1.88	3.03 ± 0.8	23.55 ± 2.37***
1	35.45 ± 3.26	7.83 ± 1.84	35.59 ± 4.48***
1.5	42.53 ± 1.78	18.58 ± 2.49	57.49 ± 1.63**
2	34.04 ± 2.22	37.97 ± 6.40	59.74 ± 1.22*
4	20.02 ± 3.32	45.09 ± 4.95	62.55 ± 0.64**
6	-	21.6 ± 1.94	39.43 ± 2.15***

Significance *** P < 0.001, **P < 0.01, *P < 0.05

## Discussion

The drug interaction studies are usually conducted in animal models to find out the mechanism before they are conducted in humans. We have selected rat as animal model since it is one of the animal, which synthesize ascorbic acid and can be maintained easily in the laboratory conditions.

Reactive oxygen species (ROS) are thought to be implicated in the pathogenesis of diabetes as well as other diseases[8]. Reactive oxygen species usually comprise radicals that have the ability to oxidize and damage DNA, proteins and carbohydrates. Hyperglycaemia appears to induce oxidative stress on cells and this can cause an increase in the production of free radicals[9]. Human antioxidant enzymes are mobilized during hyperglycaemia, but they cannot meet the continued demand due to increased oxidative stress[10]. This problem is either due to decreased intake of needed precursors or an inability to synthesise the antioxidant enzymes[11]. Antioxidant supplementation may provide the only means to reverse this process[12]. Use of typical antioxidants alone or in combination may retard or even prevent the normal progression of diabetic complications.

It was reported that L-ascorbic acid levels were decreased in diabetic patients and rats[13]. So it is felt that L-ascorbic acid supplementation may help in the treatment of diabetes mellitus. In the present study L-ascorbic acid and tolbutamide reduced blood glucose levels in normal & diabetic rats in a dose dependent manner. L-ascorbic acid when administered alone produced an early onset of action 0.5 & 1.5 h in normal and diabetic rats respectively. This early onset may be due to increase in the insulin secretion which support earlier reports that L-ascorbic acid supplementation increase the plasma insulin concentration[14]. Tolbutamide when administered in therapeutic dose produced the maximum effect at 4 h and was maintained up to 6 h in both normal and diabetic rats. In the presence of L-ascorbic acid the onset of action of tolbutamide was early and maintained for longer duration compared to tolbutamide control. Tolbutamide acts by stimulating insulin secretion (pancreatic)[15] and also by increasing tissue uptake of glucose (extra pancreatic)[16]. The early onset of action was noticed to be due to L-ascorbic acid, which was maintained later due to tolbutamide activity since both are reported to have influence on insulin secretion [14,15].

## Conclusion

The study indicates that additive action of L-ascorbic acid on pharmacodynamic response of tolbutamide may be useful to improve the tolbutamide activity in insulin resistant cases and to postpone the occurrence of diabetic complications. However further work on human patients is required to confirm the observation in diabetic condition and usefulness of L-ascorbic acid as supplemental agent for improved control of blood glucose levels when administered along with sulfonylureas.

## Competing interests

The author(s) declare that they have no competing interests.

## Authors' contributions

SS Conceived of the study, participated in the design of the study, performed the statistical analysis and drafted the manuscript.

EK Participated in the design and coordination and in the standardization of methods.

RJ Carried out the study in normal rats.

VA Carried out the study in diabetic rats.

All authors read and approved the final manuscript.

**Figure 1:**
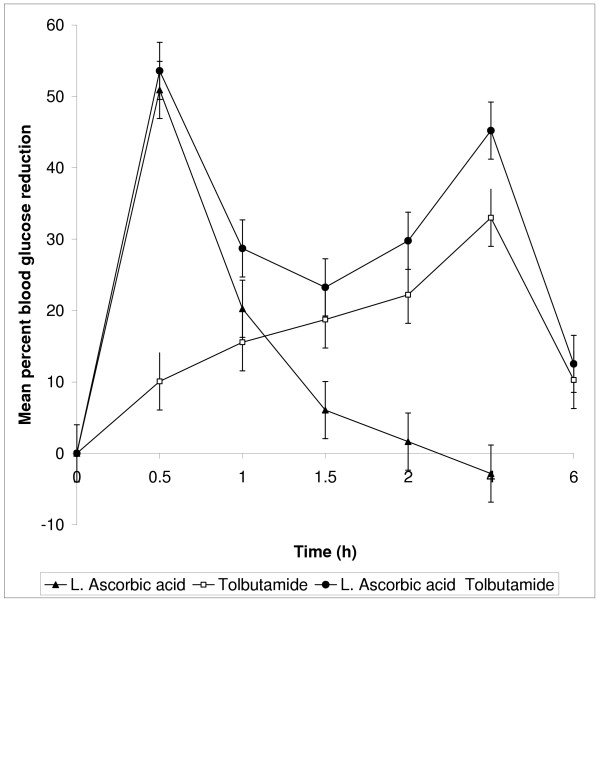
Percent Blood Glucose Reduction with L-ascorbic acid / tolbutamide / L-ascorbic acid + tolbutamide in normal rats (n = 5)

**Figure 2:**
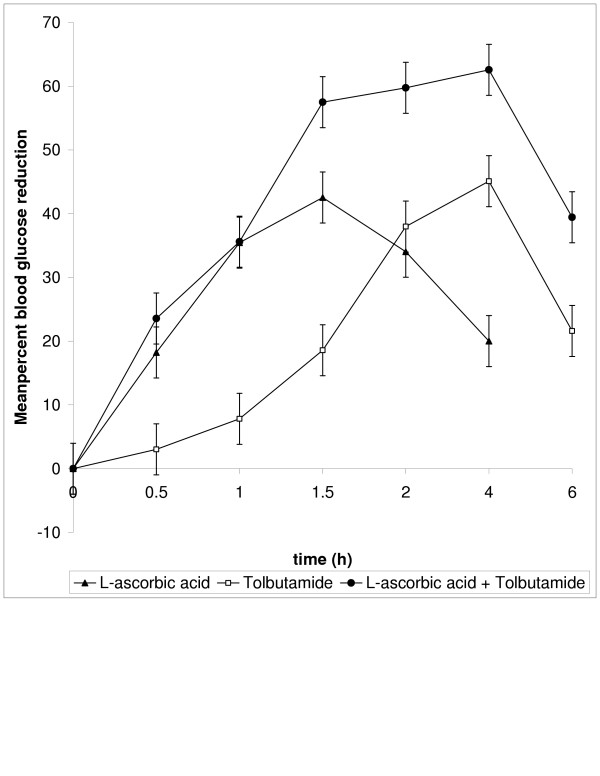
Percent Blood Glucose Reduction with L-ascorbic acid / tolbutamide / L-ascorbic acid + tolbutamide in diabetic rats (n = 5)

## Pre-publication history

The pre-publication history for this paper can be accessed here:


